# *Trichophyton mentagrophytes* Genotype VII: Sexually Transmitted Infection Beyond MSM

**DOI:** 10.1111/ajd.70144

**Published:** 2026-05-22

**Authors:** Gabriele Perego, Anna Grancini, Susanna Benardon, Stefano Ramoni, Angelo V. Marzano, Paolo Bortoluzzi

**Affiliations:** ^1^ Department of Pathophysiology and Transplantation University of Milan Milan Italy; ^2^ Central Chemical, Clinical and Microbiology Analysis Laboratory Foundation IRCCS Ca’ Granda Ospedale Maggiore Policlinico Milan Italy; ^3^ Dermatology Unit Foundation IRCCS Ca’ Granda Ospedale Maggiore Policlinico Milan Italy

**Keywords:** mycology, sexually transmitted dermatophytosis, sexually transmitted infections, tinea pubo‐genitalis, trichophyton mentagrophytes, trichophyton mentagrophytes genotype VII

Dear Editor,

*Trichophyton mentagrophytes* is a zoophilic dermatophyte causing superficial fungal infection. Among its variants, *Trichophyton mentagrophytes* genotype VII (TMVII) has been recently recognised as a distinct genotype and has been increasingly linked to sexually transmitted infections [1]. While the earliest reports of TMVII infections in Europe mainly involved patients who had had contact with commercial sex workers in Southeast Asia [2], more recent evidence indicates that TMVII is circulating across the continent, particularly among men who have sex with men (MSM) [3, 4, 5, 6].

In a previous study, we documented the first confirmed cases of sexually transmitted TMVII infection in Italy, all occurring in MSM patients and manifesting as tinea barbae. Here, we report a more recent case series collected at our Dermatology Unit between October 2024 and December 2025, which shows a more heterogeneous patient population. Epidemiological and clinical data are summarised in Table 1. The diagnosis of *T. mentagrophytes* infection was established through mycological cultures, and genotyping of the ITS1–2 region confirmed the presence of TMVII. Patients were treated with oral terbinafine 250 mg daily or itraconazole 200 mg daily for 4 weeks in combination with topical antifungal therapy. Treatment was effective in all patients, with no evidence of recurrence or antifungal resistance.

**TABLE 1:** Epidemiological, clinical features, and risk factors of our cases of sexually transmitted *Trichophyton mentagrophytes* genotype VII infection. All patients reported at least one sexual encounter within 10–30 days prior to the onset of symptoms, while none reported recent travel outside Italy or contact with animals.

Pts	Age y	Sex	Sexual behaviour	HIV+ or PrEP	Other STI	Risk factors	Sites of involvement	Clinical features
1	35	M	Heterosexual	No	No	Sexual contact with a commercial sex worker (CSW)	Groin, buttocks, pubic area and lower trunk	Highly inflammatory erythematous papules and plaques, pustules, and crusted erosive lesions
2	29	M	MSM	PrEP	NG proctitis, CT proctitis, CT pharyngitis	Multiple sexual partners	Beard, hand	Erythematous scaly lesions with raised border and pruritus
3^a^	46	M	Heterosexual	No	No	Multiple sexual partners, group sexual activities	Beard	Erythema, papules and pustules, with bacterial superinfection
4^a^	42	F	Heterosexual	No	No	Multiple partners, group sexual activities, shaving	Groin, pubic area and external genitalia	Erythematous circinate patches and inflammatory nodules
5	40	M	MSM	PrEP	MPOX, NG proctitis, NG pharyngitis, CT urethritis	Sauna	Groin, trunk, arms and legs	Erythematous, scaly, circinate patches
6	31	M	MSM	No	No	No	Beard	Erythema and pustules, with bacterial superinfection
7	27	M	Heterosexual	No	Not tested	No	Groin and pubic area	Inflammatory erythematous plaques and Majocchi granuloma, with pruritus, loco‐regional linphoadenopathies
8	30	M	NA	No	Not tested	No	Buttocks, legs, face	Erythematous‐squamous patches
9	28	M	Heterosexual	No	No	No	Beard	Granulomatous plaques and nodules
10	33	F	Heterosexual	No	CT Pharyngitis	shaving	Buttocks and pubic area	Highly inflammatory erythematous papules and plaques
11	36	M	Heterosexual	No	No	CSW	Beard and neck	Erythematous, scaly, circinate patches
12	29	F	Heterosexual	No	No	No	Pubic area and lower trunk	Erythematous and scaly patches
13	24	M	MSM	PrEP	NG proctitis, NG pharyngitis	Shaving, sauna	Groin, buttocks and beard	Erythema and pustules

^a^ Patient 3 and 4 were partners. MSM, men who have sex with men; NG, *Neisseria gonorrhoeae* ; CT, *Chlamydia trachomatis* ; PrEP, Pre‐Exposure Prophylaxis for HIV infection.

Following the first reports of autochthonous sexually transmitted TMVII infections in France [3, 4] and Italy [5], increasing evidence has documented the spread of this infection across several European countries and worldwide [2]. While early reports described cohorts composed exclusively of men who have sex with men (MSM) [3, 4, 5, 6], subsequent studies have identified TMVII infections in heterosexual individuals of both sexes [1, 7, 8]. In our cohort, more than 60% of patients (8/13) reported heterosexual orientation and about 23% (3/13) were women.

TMVII is not restricted to a specific sexual related anatomical site (e.g., beard or pubo‐genital area) but may involve multiple body regions, such as arms and legs [1]. Many of our patients showed atypical presentations of tinea corporis, including painful erythematous plaques, pustules and nodules, occasionally progressing to granulomatous lesions or erosive lesions with eschar formation [2]. Although TMVII is transmitted through anthropophilic contact, its close genetic relationship with zoophilic genotypes within the *T. mentagrophytes* complex may account for the marked inflammatory response observed [7]. Interestingly, more inflammatory lesions predominantly involved hair‐bearing areas, such as the beard and pubic region, whereas lesions on the trunk or shaved perigenital skin more often displayed the classical ringworm morphology. Two patients in our cohort were stable partners presenting with matching lesions (tinea cruris in the female partner and tinea barbae in the male partner) following reported oral–genital contact, further supporting sexual transmission as a likely route of infection [9].

Four of 13 patients presented with concomitant sexually transmitted infections (STIs), underscoring the importance of routine screening for other STIs in patients with fungal infections suspected to be sexually transmitted [1]. One patient concurrently showed cutaneous manifestations of both TMVII infection and mpox (Figure 1d). Mixed cutaneous lesions caused by different conditions should therefore be considered, as they may complicate clinical assessment [2]. No obvious STI risk factors were identified in 6 of 13 cases: the hypotheses considered include indirect environmental contamination, direct human‐to‐human transmission through non‐sexual contact, and unreported heterosexual or same‐sex sexual relationships.

**FIGURE 1:**
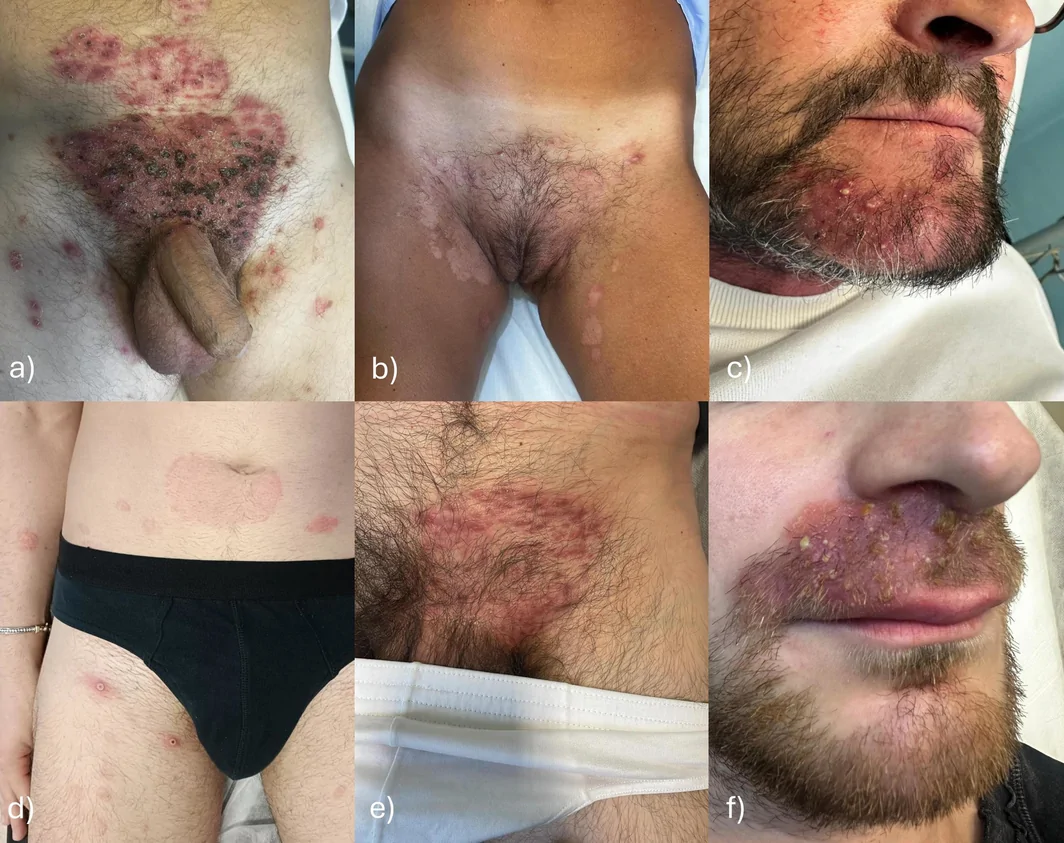
Clinical features of sexually transmitted *Trichophyton mentagrophytes* genotype VII infection. Specifically: (a) inflammatory erythematous plaques and crusted erosive lesions; (b) tinea pubis and tinea cruris in a female patient (patients in panels b and c are partners); (c, f) tinea barbae with pustules and signs of secondary bacterial infection (honey‐coloured crusts), confirmed by microbiological swab; (d) concomitant lesions of TMVII tinea corporis and mpox; (e) tinea pubis with granulomatous features.

The diagnosis of sexually transmitted TMVII infection relies on a high index of clinical suspicion and molecular confirmation through PCR amplification of the ITS1–2 region. However, this test is not universally available and is not routinely performed, suggesting that the number of confirmed cases may underestimate the true burden of this infection [10]. Supporting this hypothesis, in a retrospective study Maci et al. reported 107 cases of sexually transmitted dermatophytosis (STD) diagnosed at their dermatology clinic in Milan between 2010 and 2023, with half of the cases occurring in 2022–2023 alone [11].

In conclusion, TMVII infection is increasingly recognised as an emerging sexually transmitted infection (STI) with a growing global distribution. Unlike other emerging strains causing tinea genitalis, such as T. Indotinae, no specific antifungal resistance has been reported for TMVII [9]. Dermatologists should maintain clinical awareness, as it may affect the broader sexually active population.

## Funding

The authors have nothing to report.

## Ethics Statement

The authors have nothing to report.

## Conflicts of Interest

The authors declare no conflicts of interest.

## Data Availability

The data that support the findings of this study are available from the corresponding author upon reasonable request.
